# Supplementary material for: Flow Dynamics through a High Swelling Nanofiber Membrane Processed at Different Relative Humidities: A Study on a FexOy/Polyvinyl Alcohol Composite
Source: Membranes (Basel). 2024 Aug 30;14(9):189. doi: 10.3390/membranes14090189 (PMC11433868; doi:10.3390/membranes14090189)
Supplement: Supplementary file 1 [file membranes-14-00189-s001.zip › membranes-3138667-supplementary.pdf]

Transmission electron microscopy (TEM) was performed with a MET Zeiss 109 (Oberkochen, Germany) operating at 120 kV and the images were taken with a Gatan W10000 camera. The samples were prepared by electrospinning the PVA + IONPs solution 20 seconds over Cu grids.

Figure S1 shows different TEM images of PVA + IONPs nanofibers, where a good dispersion of IONPs inside the nanofiber can be found. Although the images were not obtained using high-resolution TEM (HRTEM), an estimation of the IONPs size distribution was performed using the open-source software ImageJ (1.51w, Wayne Rasband, National Institutes of Health, Stapleton, NY, USA). The distribution shows a most probable value at (3 ± 1) nm and an average diameter of (7.8 ± 5.3) nm. The RH of the electrospinning chamber does not introduce significant changes in the TEM images considering the IONPs presence and dispersion.

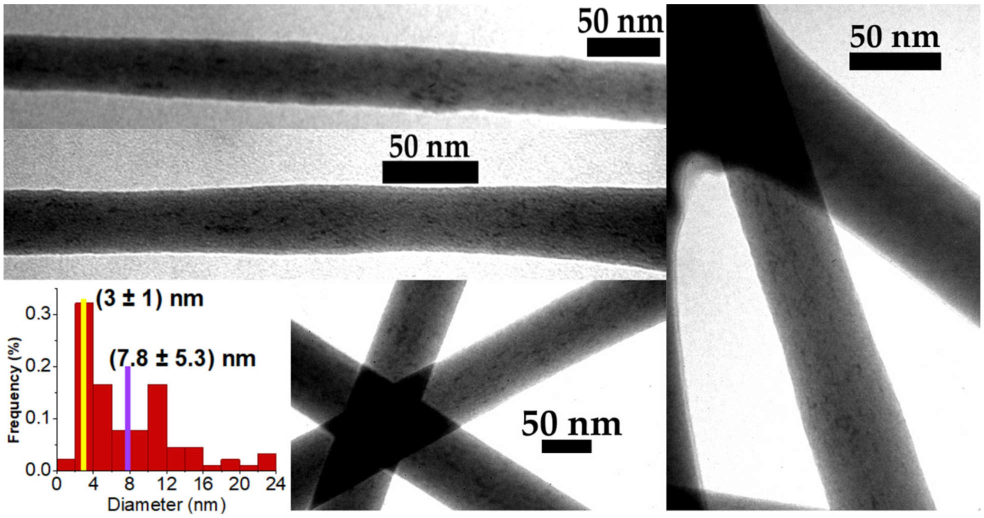

Figure S1. TEM images of PVA + IONPs electrospun membranes.

S2. FTIR Characterization

Spectra were recorded with a Perkin Elmer (Spectrum Two Perkin Elmer, Shelton, CT, USA), in the range 4000 cm^{-1} to 450 cm^{-1} with a resolution of 4 cm^{-1}.

ATR-FTIR spectroscopy was used to determine the changes in the chemical structure of the PVA membranes due to the incorporation of IONPs. The infrared spectra of the different materials are shown in Figure S2A. A detail of the same is given in Figure S2B. As can be seen, the membranes show the bands characteristic of PVA under both intermediate and low humidity conditions of the fabrication process. Bands were observed around 3320 cm^{-1} and 1095 cm^{-1}, corresponding to the stretching vibrations of the O-H and C-O bonds, respectively. The bands at 2943 cm^{-1} and 2908 cm^{-1} are associated with the asymmetric and symmetric stretching vibrations of the C-H bond of the CH_2 group, while the band at 1446 cm^{-1} is attributed to the bending vibrations of the C-H bond. In addition, a band at 850 cm^{-1} corresponding to CH_2 rocking in the PVA.

In the PVA + IONPs spectra, the O-H stretching is shifted to lower wavenumbers (Figure S2B). This shift has been attributed by some authors to the formation of hydrogen bonds between the IONPs and the hydroxyl groups of PVA [1–3]. These results confirm the IONPs presence in the PVA + IONPs nanofibers.

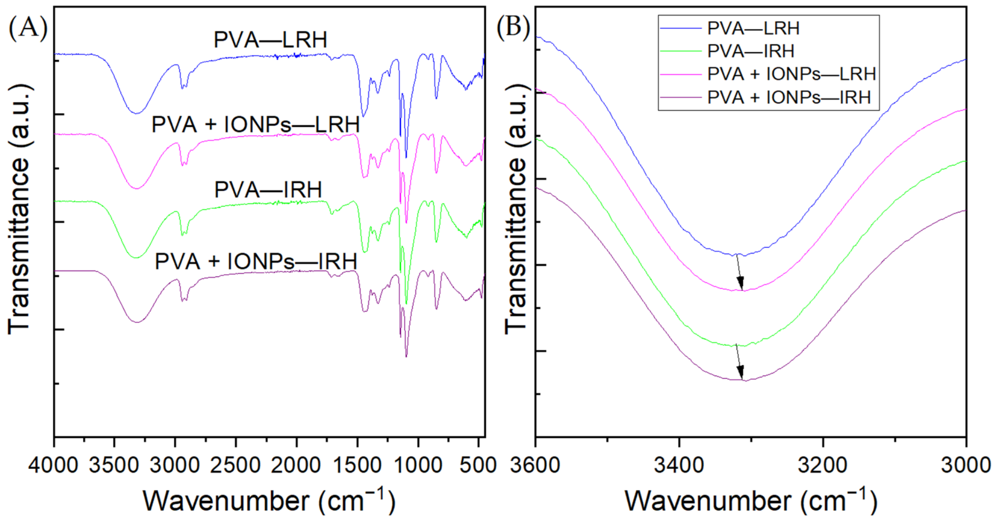

Figure S2. (**A**) FTIR spectra of PVA and PVA + IONPs at the different RHs studied. (**B**) Close-up showing a shift in the O-H stretching.

S3. XRD Characterization

X-ray diffraction data was obtained with a Phillips PW3710 (Almelo, The Netherlands) diffractometer operated with Cu-Kα radiation and a graphite monochromator. The measurements were made from 5 to 80° (2θ) with a step of 0.026° and time per step was 1200 s at RT. Figure S3 shows the comparison of the diffraction patterns between PVA and PVA + IONPs membranes. The RH of the electrospinning chamber does not introduce significant changes in the XRD patterns. It is observed that the IONPs presence introduce new peaks associated with two different iron oxides: lepidocrocite (2θ = 27.8°) and goethite (2θ = 32.5°, 34.6°, 39.3° and 50.8°) [4]. These results also confirm the IONPs presence in the PVA + IONPs membrane.

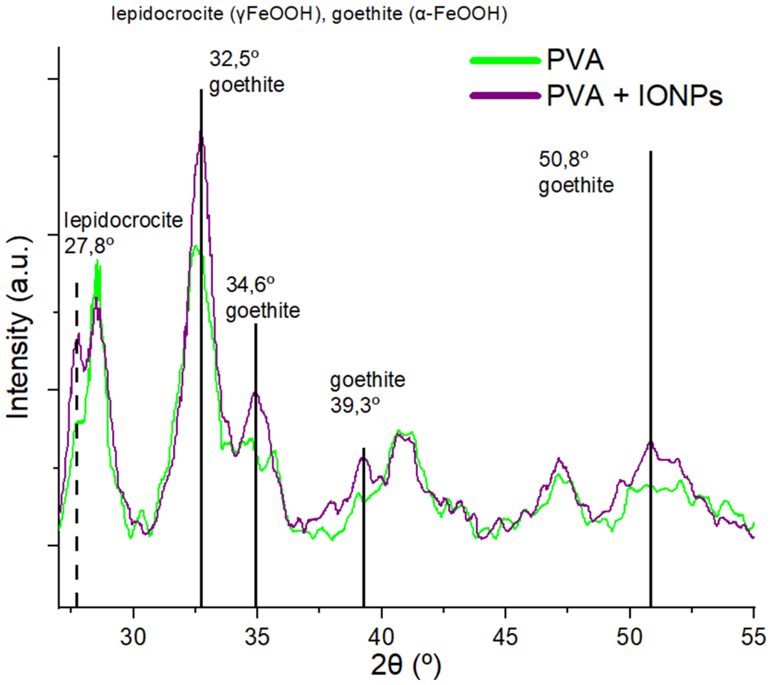

Figure S3. XRD diffraction patterns of PVA and PVA + IONPs.

References

1. Torasso, N.; Vergara-Rubio, A.; Pereira, R.; Martinez-Sabando, J.; Baudrit, J.R.V.; Cerveny, S.; Goyanes, S. An in Situ Approach to Entrap Ultra-Small Iron Oxide Nanoparticles inside Hydrophilic Electrospun Nanofibers with High Arsenic Adsorption. *Chem. Eng. J.* **2023**, *454*, 140168. https://doi.org/10.1016/j.cej.2022.140168.
2. Kayal, S.; Ramanujan, R. V. Doxorubicin Loaded PVA Coated Iron Oxide Nanoparticles for Targeted Drug Delivery. *Mater. Sci. Eng. C* **2010**, *30*, 484–490, doi:10.1016/J.MSEC.2010.01.006.
3. Lee, J.; Isobe, T.; Senna, M. Preparation of Ultrafine Fe3O4Particles by Precipitation in the Presence of PVA at High PH. *J. Colloid Interface Sci.* **1996**, *177*, 490–494, doi:10.1006/JCIS.1996.0062.
4. Ahn, T.; Kim, J.H.; Yang, H.M.; Lee, J.W.; Kim, J.D. Formation Pathways of Magnetite Nanoparticles by Coprecipitation Method. *J. Phys. Chem. C* **2012**, *116*, 6069–6076, doi:10.1021/jp211843g.
